# The Danger of the Dead Tooth

**Published:** 1920-05-15

**Authors:** 


### "THE DANGER OF THE DEAD TOOTH."

With remarkable frequency the public has brought prominently to its notice one or other of the evil consequences likely to arise from dental neglect and indifference to any condition of the teeth, unless it is accompanied by obvious and insistent toothache. But there are regrettably few people among our millions, and practically none among the poorer population, who follow the very excellent course of having periodically a thorough dental examination.

### The Meaning of Pain.

The absurdity of assuming that pain is the particular indication which should prompt a visit to the dentist is too obvious to be dealt with here, and some recent notes in the daily press, coming from the pens of those well qualified to speak, may have done something to impress it upon their readers in the morning train. But a "dead tooth" is, to the uninitiated, something of a vague term, and so is the oft-repeated "oral sepsis." If one may judge from the dental condition too frequently noted in the middle-aged person who protests that his teeth are excellent and who receives any suggestion as to sepsis in his mouth as utterly ridiculous, then the vast majority even of educated people have not the slightest conception of what these local troubles imply, and no amount of journalistic exhortation will bring them to the examining chair if its scheme leaves decision as to the necessity in the patient's own hands. Periodic examination by the expert is the only rational and satisfactory procedure, and let this be urged to the utmost. It will pay both the individual and the nation.

### The Power of Septic Foci.

We have often heard it explained, in argument against the potential power of sepsis arising in the mouth, that there are thousands of people who have for years an appallingly bad dental condition, and yet suffer apparently no constitutional ill-effects whatever. Now the arguments in defence of the medical warnings are well known to professional readers of these pages, and they are among the strongest that can be brought to bear. Recently, however, the results of some experiments came to our notice which, although as yet unpublished in detail, throw considerable light on the oral sepsis question. The essential point of the situation is that, admitting the unavoidable ingestion of all manner of bacteria from pockets of decayed teeth, from chronically discharging inflammations of the gums, or from any of the possible types of septic foci in the mouth, the following questions arise. Do these bacteria, of which the *streptococcus pyogenes longus* is the most virulent, pass the gastric barrier? Do they survive their passage through the gastric juice, and so reach the definitely absorptive part of the system? Briefly, by a new experimental method cultures from the duodenal contents at any particular time can be made, and it is found that virulent streptococci pass through only when deficient gastric secretion is present. Gastric deficiency, temporary or chronic, is perhaps one of the earliest signs of constitutional decline. As it begins, so the septic organisms start to invade the whole alimentary canal. The bacteria are, as it were, waiting at the door for some lapse in general health to let them in. Once a passage is established a typical vicious circle begins, and the doctor meets with a case where, though the dental sepsis may not be obviously severe, its removal produces an almost spectacular cure.

### Children and Growth.

This simple sketch has been applied mainly to the adult, but, allowing for essential differences, we can equally well apply its moral to the child. Slow septic absorption from any focus, be it from the mouth or elsewhere, produces in the young that retardation of growth, that pale-faced anæmic condition, a lack of lustre in the eyes, the tired sleepy look, and general damaging influence, which recent propaganda has done much to bring to the mother's notice. Nothing could be more urgent than the problem of dental caries among the schoolchildren of this country.

The stomach of the labourer with the foully septic mouth is more difficult to disturb, but if for any reason his vitality falls, then, as was repeatedly exemplified in the war period, the result is correspondingly more severe. To the professional reader we would admit that we have passed by the other method of aseptic extension, namely, by lymphatic paths, and typified by the adenitis, the swollen glands of the child; but so urgent is the call for better care of the nation's teeth, and the necessity for voluntary periodic dental examination, that we venture to draw attention to one small aspect of the question.

